# Preterm birth, bullying victimization, and mental health in adulthood: A prospective cohort study in Germany

**DOI:** 10.1111/camh.70025

**Published:** 2025-08-08

**Authors:** Yanyan Ni, Nicole Tsalacopoulos, Peter Bartmann, Dieter Wolke

**Affiliations:** ^1^ Department of Psychology University of Warwick Coventry UK; ^2^ School of Public Health University of Hong Kong Hong Kong China; ^3^ Department of Population Health Sciences University of Leicester Leicester UK; ^4^ Turner Institute for Brain and Mental Health, School of Psychological Sciences Monash University Melbourne Vic. Australia; ^5^ Department of Neonatology and Pediatric Intensive Care University Hospital Bonn Bonn Germany; ^6^ Division of Health Sciences, Warwick Medical School University of Warwick Coventry UK

**Keywords:** VP/VLBW, bullying victimization, mental health, mediation, moderation

## Abstract

**Background:**

To examine the moderating and mediating roles of bullying victimization in the association between preterm birth and mental health in adulthood.

**Method:**

As part of a prospective geographically defined longitudinal study in Germany, 260 adults born very preterm (<32 weeks of gestation) and/or with very low birth weight (birth weight < 1500 g; VP/VLBW) and 229 term‐born controls were assessed at the 26‐year follow‐up. Bullying victimization was reported by parents at 8 and 13 years. At age 26, internalizing symptoms were reported via questionnaire by both participants and parents, and diagnoses for mood and anxiety disorders were obtained via structured interviews. Associations were analyzed using adjusted negative binomial regression and robust Poisson regression models.

**Results:**

We found associations of VP/VLBW birth with internalizing problems in adulthood (adjusted incidence rate ratio (IRR) range: 1.43–2.02). Across both preterm and term‐born groups, being bullied, in particular, chronically (more than one time point) was associated with increased internalizing symptoms (adjusted IRR range: 1.27–1.69). Across both groups, bullying victimization at two time points was also associated with increased risk of mood disorders (adjusted IRR 2.08, 95% CI 1.27–3.42). Bullying victimization mediated 15.8% of the effects of VP/VLBW birth on self‐reported internalizing symptoms and 8.5% on parent‐reported internalizing symptoms. Bullying victimization did not moderate the associations between VP/VLBW birth and mental health outcomes in adulthood.

**Conclusions:**

Our findings suggest that being bullied may have adverse effects on mental health in both VP/VLBW and term‐born children that last into adulthood. The association between VP/VLBW birth and internalizing symptoms was partly mediated by bullying victimization in childhood and adolescence.

**Key practitioner message**

**What is known?**

- Both bullying victimization and preterm birth affect long‐term mental health outcomes.
- It remains unclear whether bullying victimization moderates or mediates the relationship between VP/VLBW birth and mental health in adulthood.

**What is new?**

- Bullying victimization could have adverse effects on mental health in both VP/VLBW and term‐born children that last into adulthood.
- VP/VLBW children were bullied more often in childhood and adolescence than term‐born children, and this partly explained that they had more internalizing symptoms.

**What is significant for clinical practice?**

- Our findings emphasize the need for interventions and support to help reduce peer bullying, which may reduce the burden of mental illness in adults in both preterm and general populations.

## Introduction

Both bullying victimization and preterm birth affect mental health in adulthood (Anderson et al., 2021; Moore et al., 2017). Bullying involves intentional and repeated acts of aggression over time with an imbalance of power (Olweus, 1994). In 2018, 30% of the 15‐year‐olds globally experienced frequent victimization and over 20% in European countries (Hosozawa et al., 2021). Victims of peer bullying in childhood are at elevated risk for depression and anxiety in adulthood (Moore et al., 2017; Wolke & Lereya, 2015). However, most of the research on bullying has focused on general populations of children and young people, with little attention given to the experiences of those biologically at risk owing to preterm birth. Preterm birth is associated with shorter stature, motor and cognitive disabilities, internalizing problems, and social difficulties in childhood and adolescence (Ni et al., 2020; Ritchie, Bora, & Woodward, 2015; Saigal & Doyle, 2008; Wolke, Johnson, & Mendonça, 2019). These factors may put preterm children at increased risk to become targets for bullies, and several studies reported that very preterm (VP; <32 weeks of gestation) and/or very low birth weight (VLBW; birth weight < 1500 g) children are at elevated risk of being bullied by peers in childhood and adolescence (Day et al., 2015; Grindvik et al., 2009; Nadeau, Tessier, Lefebvre, & Robaey, 2004; Wolke, Baumann, Strauss, Johnson, & Marlow, 2015; Yau et al., 2013).

**Figure 1:**
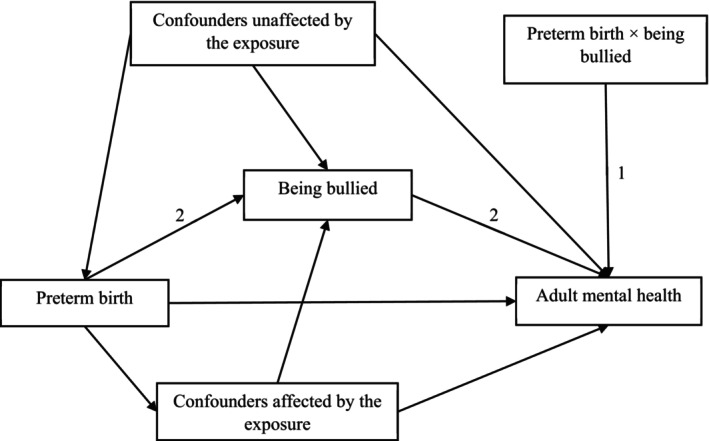
Conceptual models showing the relationship between preterm birth, bullying victimization, and mental health in adulthood. Pathway 1 tested the moderation effect of bullying victimization in childhood in the association between preterm birth and mental health in adulthood through interaction terms; pathway 2 tested the mediation effect of bullying victimization in the association between preterm birth and mental health in adulthood. Confounders unaffected by the exposure (i.e., preterm birth) included sex, multiple birth, and family socioeconomic status at birth. Confounders affected by the exposure (i.e., preterm birth) include pre‐existing internalizing symptoms at age 6, and neurosensory impairment in childhood.

There are two potential mechanisms of how preterm birth and being bullied at school age may affect long‐term mental health outcomes (Figure 1). First, preterm‐born individuals may be more vulnerable to the negative effects of bullying victimization due to perinatal adversity, compared with term‐born peers. Thus, bullying victimization would have more adverse effects on mental health in preterm‐born than term‐born individuals (moderation effect). This is supported by the diathesis‐stress model in which the emergence of mental health disorders depends on the combination of an individual's inherent vulnerability for developing the disorder and personal experience of stressful events (Broerman, 2017). There is some empirical evidence that individuals born extremely low birth weight (ELBW; birth weight < 1000 g) are more vulnerable to childhood adversity (e.g., peer victimization, physical or sexual abuse, family dysfunction) compared with peers born normal birth weight (NBW) (Van Lieshout et al., 2018; Wolke, 2018). Vulnerability to bullying victimization may, however, depend on the areas of outcomes examined (Day et al., 2017). For instance, in one study, bullying victimization appeared to affect ELBW adults more than controls in some outcomes, such as crime conviction and chronic health conditions (Day et al., 2017), but for other areas of social functioning (e.g., the likelihood of having offspring, family functioning, loneliness, social support, self‐esteem, income) (Day et al., 2017), bullying victimization affected ELBW and term‐born equally. However, bullying victimization was assessed retrospectively in this study, and prospective evidence on whether bullying victimization in childhood moderates the association between prematurity and adult outcomes is lacking.

Alternatively, preterm‐born individuals develop more internalizing symptoms and mental health disorders in adulthood due to being bullied more often than their term‐born peers (mediation effect). A study of two prospective cohorts of VP or extremely preterm (EP; <26 weeks of gestation) school children reported that those who were bullied, in particular, across two time points reported more emotional problems 5–7 years later compared with term‐born children (Wolke et al., 2015). More recent evidence suggests a significant mediation path for VP/EP birth and psychosis via bullying victimization (Liu et al., 2019). However, it is unknown whether bullying victimization mediates the relationships between preterm birth and adult mental health outcomes (Anderson et al., 2021).

The aims of this prospective study were to investigate, first, whether VP/VLBW birth and being bullied at school age are individually associated with more internalizing symptoms and increased risk for mood and anxiety disorders in adulthood. Second, to test two alternative mechanisms, that is, whether bullying victimization moderates or mediates the relationships between VP/VLBW birth and mental health outcomes in adulthood.

## Methods

### Participants

The Bavarian Longitudinal Study is a prospective whole population study of children born in a geographically defined area of Southern Bavaria (Germany) between January 1985 and March 1986 who required admission to one of 16 children's hospitals within the first 10 days after birth. The enrollment and data collection procedures for the BLS have been described in detail elsewhere (Wolke & Meyer, 1999). In total, 682 individuals were born very preterm (VP; <32 weeks of gestation) and/or with very low birth weight (VLBW; <1500 g). At 26 years of age, 411 were alive and eligible for inclusion and 260 (63.3%) were assessed. The assessed VP/VLBW sample in adulthood did not differ from the nonassessed sample in terms of gestational age, birth weight, duration of hospitalization, gender, maternal age, parental marital status, and childhood cognitive scores, but had fewer prenatal complications and were of higher socioeconomic status (Eryigit Madzwamuse, Baumann, Jaekel, Bartmann, & Wolke, 2015).

**Figure 2:**
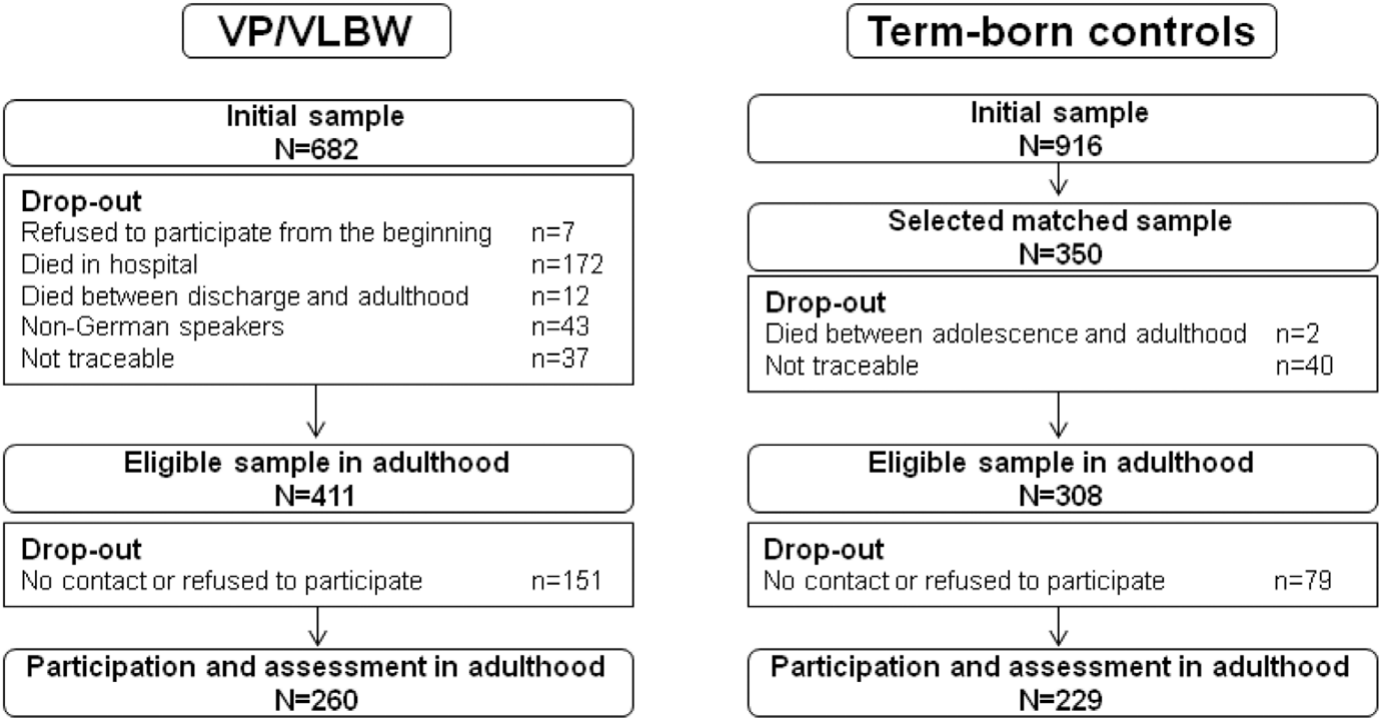
Eligible sample of VP/VLBW and term‐born control participants at age 26 years

Healthy term‐born comparisons were recruited from the same obstetric hospitals at birth. Of the initial 916 control children alive at 6 years, 350 were randomly selected to be comparable to the overall distribution of child sex, family socioeconomic status (SES), and maternal age of the VP/VLBW group. At 26 years of age, 308 participants were eligible for inclusion and 229 (74.4%) were assessed (Figure 2).

Initial ethical approval was obtained from the Ethics Committee of the University of Munich Children's Hospital and the Bavarian Health Council. Ethical approval for the follow‐up in adulthood was obtained from the Ethical Board of the University Hospital Bonn (reference 159/09). Parents provided informed written consent within 48 hr of their child's birth, and all participants provided informed written consent for the assessments in adulthood. In case of severe impairment of the adult participant, consent was provided by an assigned guardian (usually a parent). The STROBE guidelines were used to ensure the reporting of this cohort study (Table S1).

## Measures

### Bullying victimization in childhood and adolescence

Bullying victimization at age 8 years was assessed via a structured parent interview. Parents were asked whether their child had been a victim of bullying by peers in the last 6 months, using two items: (a) being insulted, teased, or bullied by peers; or (b) being beaten up by peers. Children who were being bullied ‘several days per month’ to ‘every day’ were considered to have been bullied. At age 13 years, bullying victimization was assessed using one item of the Strengths and Difficulties Questionnaire (SDQ) (Goodman, 2001) completed by both participants and their parents on a 3‐point scale: being picked on or bullied. The child was considered to have been bullied if either the child or the parent answered ‘certainly true’ or ‘somewhat true’. We previously reported that there was fair to moderate agreement between parent‐reports and self‐reports at 13 years (kappa = .39) and there was a significant association between parent‐reports and self‐reports using McNemar test (*p* = .012) (Ni, Baumann, & Wolke, 2024). A three‐category bullying variable was further constructed: (1) 2 = bullying victimization at two time points (age 8 and 13 years); (2) 1 = bullying victimization at one time point (either age 8 or 13 years); and (3) 0 = no bullying victimization at either age.

### Mental health in adulthood

Internalizing problems in adulthood were assessed with the Achenbach Young Adult Self‐Report (Achenbach, 1997). In addition, parents rated their child's internalizing symptoms using the Young Adult Behavior Checklist (Achenbach, 1997). For both instruments, each item was rated on a scale from 0 (*not true*) to 2 (*very/often true*) and then added up into a total score, with higher scores indicating more internalizing symptoms. We focused on internalizing problems rather than externalizing problems, as previous research indicates that compared with term‐born individuals, those born VP/VLBW experience more internalizing problems but are not at increased risk for externalizing, rule‐breaking, intrusive, and antisocial personality problems (Pyhala et al., 2017).

Mood and anxiety disorder diagnoses were obtained according to DSM‐IV criteria using a computer‐assisted version of the Munich Composite International Diagnostic Interview (DIA‐X/M‐CIDI) that assessed symptoms in the last 6 months before the interview (Wittchen et al., 1995; Wittchen & Pfister, 1997). Our definitions of any anxiety or mood disorders included all DSM‐IV defined subtype diagnoses, which have been previously described (Jaekel, Baumann, Bartmann, & Wolke, 2018). Details on this are provided in Table S2. The DIA‐X/M‐CIDI is a fully structured clinical interview that was supplemented by a separate respondent booklet including cognitive aids to assist with answering questions about symptom onset, duration, recency, and severity (Carter, Wittchen, Pfister, & Kessler, 2001). The DIA‐X/M‐CIDI DSM‐IV main diagnostic categories' test–retest reliability (kappa = .56–.81) (Wittchen, Lachner, Wunderlich, & Pfister, 1998) and procedural validity (kappa *= *.50–.96) (Reed et al., 1998) have been reported to be acceptable. The interviews were conducted by trained psychologists. Regarding reliability, every 2 months, we conducted refresher meetings for the interviewers, during which we listened to a recording of an interview. The interviewers were trained to achieve a kappa value exceeding .80, and any discrepancies were discussed. This approach was deemed more beneficial than mere reliability sessions. In instances of uncertainty regarding coding throughout the study, a designated head psychologist provided guidance and addressed any queries.

### Potential confounders

Gestational age at birth was determined from maternal reports of the last menstrual period and serial ultrasounds during pregnancy. Birth weight was recorded from birth records.

The choice of potential confounders was based on previous research (Analitis et al., 2009; Wolke et al., 2015), including sex, multiple births, family SES (obtained via standardized interview neonatally and computed as a weighted composite score of parents' education and occupation and grouped as low, middle, and high) (Bauer, 1988), preexisting internalizing problems in childhood at 6 years (assessed with the Child Behavior Checklist) (Achenbach, 1991), and neurosensory impairment in childhood (defined as having cognitive impairment (childhood IQ < 70), nonambulatory cerebral palsy, visual impairment, or hearing impairment).

### Statistical analyses

Data were analyzed in STATA 17.0 and R 4.2.1. Differences between VP/VLBW and term‐born individuals were tested using *t*‐tests or Wilcoxon rank‐sum tests for continuous variables and Chi‐square tests for categorical variables. Associations of bullying victimization and VP/VLBW birth with self‐reported and parent‐reported internalizing symptom scores were analyzed using negative binomial regression given the skewed distribution and the overdispersion in the outcomes (model fit statistics reported in Table S3) (Hilbe, 2011). Associations with any mood disorder diagnosis (yes vs. no) and any anxiety disorder diagnosis (yes vs. no) were analyzed using Poisson regression with robust standard errors for model estimates (model fit statistics reported in Table S4). Robust Poisson regression models are a widely used approach for common binary outcomes (prevalence of mood and anxiety disorders range 17.5%–26.5% in our study). Incidence rate ratio (IRR) and 95% confidence interval (CI) were estimated. We adjusted for sex, multiple birth, family SES at birth, preexisting internalizing symptoms at age 6, and neurosensory impairment in childhood when analyzing the associations between bullying victimization and adult mental health, but only adjusted for sex, multiple birth, and family SES at birth when analyzing the associations between VP/VLBW birth and adult mental health (Figure 1). Additionally, we tested moderation effects through adding interaction terms between prematurity and bullying victimization. Multiple imputations were used to impute missing values in bullying victimization and confounders (Sterne et al., 2009). The mi impute chained command was used when performing association analyses in STATA. Imputation models were based on the missing at random assumption, and 20 imputed datasets were created.

We performed a causal mediation analysis to analyze the mediating role of bullying victimization in the associations between VP/VLBW birth and mental health outcomes using the R package CMAverse (Shi, Choirat, Coull, VanderWeele, & Valeri, 2021). For this part of the analysis, we separated confounders to those affected and unaffected by the exposure (VP/VLBW birth). The former included preexisting internalizing symptoms at age 6 and neurosensory impairment in childhood, and the latter included sex, multiple birth, and family SES at birth. Confounders affected by the exposure complicated the analysis as they were part of the causal pathway between the exposure and the outcome. Simply adjusting for them together with confounders unaffected by the exposure could lead to inaccurate estimation of causal effects and introduce bias in the mediation analysis. Given the existence of mediator‐outcome confounders affected by the exposure, we adopted a g‐formula approach to estimate the mediation effects of bullying victimization with standard errors estimated through bootstrapping (Shi et al., 2021). The g‐formula approach can estimate the direct and indirect effects of an exposure on an outcome while properly accounting for confounders that are either affected or unaffected by the exposure. The mediator was modeled using multinomial regression and outcomes were modeled using negative binomial regression (model fit statistics reported in Table S5). We performed sensitivity analyses for unmeasured confounding via the E‐value approach to assess the robustness of mediation results (Smith & VanderWeele, 2019; VanderWeele & Ding, 2017). The E‐value is defined as the minimum strength of association, on the risk ratio scale, that an unmeasured confounder would need to have with both the exposure and the outcome to fully explain away a specific exposure–outcome association, conditional on the measured covariates. We specified the option “multimp = TRUE” in the CMAverse package to perform multiple imputations and 20 imputed datasets were created.

## Results

In total, 413 participants had data on bullying victimization in childhood, 460 had data on self‐reported internalizing symptoms in adulthood, 431 on parent‐reported internalizing symptoms, and 486 on mood and anxiety disorders. By definition, VP/VLBW adults had lower gestational age and birth weight compared with term‐born controls. The two groups significantly differed in proportions of multiple births, family SES, and childhood neurosensory impairment (*p* < .05; Table 1). There were no significant group differences in sex and preexisting internalizing problems (*p* > .05; Table 1). Significantly more VP/VLBW participants than term‐born participants reported bullying victimization at two time points in childhood (23.7% vs. 12.1%, *p* < .05; Table 1).

**Table 1:** Sample descriptives

		VP/VLBW, *N* = 260	Controls, *N* = 229	*p*‐Value
Neonatal and family characteristics at birth/5 months				
Gestation (weeks)	Mean (*SD*)	30.6 (2.2) [*n* = 260]	39.7 (1.2) [*n* = 229]	<.001
Birth weight (g)	Mean (*SD*)	1323.5 (316.5) [*n* = 260]	3363.5 (444.9) [*n* = 229]	<.001
Multiple birth	% (*n*/*N*)	26.5% (69/260)	3.1% (7/229)	<.001
Male	% (*n*/*N*)	53.1% (138/260)	46.7% (107/229)	.161
Intraventricular hemorrhage (stage 3/4)	% (*n*/*N*)	7.7% (20/260)	–	–
Bronchopulmonary dysplasia at day 28	% (*n*/*N*)	52.7% (137/260)	–	–
Socioeconomic status at birth				
High	% (*n*/*N*)	20.5% (53/259)	33.6% (77/229)	.003
Middle	% (*n*/*N*)	47.1% (122/259)	42.8% (98/229)	
Low	% (*n*/*N*)	32.4% (84/259)	23.6% (54/229)	
Development outcomes in childhood				
Childhood neurosensory impairment	% (*n*/*N*)	24.1% (58/241)	0.4% (1/227)	<.001
Preexisting internalizing problems at age 6	Mean (*SD*)	7.5 (5.5) [*n* = 239]	6.7 (4.7) [*n* = 229]	.099
Preexisting internalizing problems at age 6	Median (P25, P75)	6.0 (3.0, 11.0)	6.0 (3.0, 10.0)	.194
Bullying victimization in childhood				
No	% (*n*/*N*)	38.2% (71/186)	51.6 (115/223)	.003
Being bullied at one time point	% (*n*/*N*)	38.2% (71/186)	36.3% (81/223)	
Being bullied at two time points	% (*n*/*N*)	23.6% (44/186)	12.1% (27/223)	
Outcomes in adulthood				
Self‐reported internalizing symptoms	Mean (*SD*)	9.4 (8.3) [*n* = 234]	6.6 (6.0) [*n* = 226]	<.001
Self‐reported internalizing symptoms	Median (P25, P75)	7.0 (3.0, 14.0)	5.0 (2.0, 10.0)	.001
Parent‐reported internalizing symptoms	Mean (*SD*)	6.8 (6.3) [*n* = 224]	3.6 (3.4) [*n* = 207]	<.001
Parent‐reported internalizing symptoms	Median (P25, P75)	5.0 (2.0, 11.0)	2.0 (1.0, 6.0)	<.001
Any mood disorder diagnosis	% (*n*/*N*)	22.6% (58/257)	17.5% (40/229)	.162
Any anxiety disorder diagnosis	% (*n*/*N*)	26.5% (68/257)	24.9% (57/229)	.693

### VP/VLBW birth and mental health in adulthood

VP/VLBW birth was significantly associated with an increased risk of both self‐ and parent‐reported internalizing problems in adulthood (Table 2). The associations remained significant (self‐reported: IRR 1.57, 95% CI 1.30–1.90; parent‐reported: IRR 2.02, 1.66–2.46) after adjusting for sex, multiple birth, family SES at birth, preexisting internalizing symptoms at age 6, and neurosensory impairment in childhood. No significant associations were found for diagnoses of mood and anxiety disorders (Table 2).

**Table 2:** Associations of VP/VLBW birth and bullying victimization with adult mental health

	
	Unadjusted	Adjusted
	IRR (95% CI)	IRR (95% CI)
Self‐reported internalizing symptoms [*N* = 460]		
VP/VLBW birth (ref. = term‐born)	**1.43 (1.20, 1.70)**	**1.57 (1.30, 1.90)**
Bullying victimization (ref. = no victimization)		
Being bullied at one time point	**1.27 (1.03, 1.56)**	1.20 (0.98, 1.47)
Being bullied at two time points	**1.69 (1.31, 2.17)**	**1.61 (1.26, 2.07)**
Parent‐reported internalizing symptoms [*N* = 431]		
VP/VLBW birth (ref. = term‐born)	**1.86 (1.55, 2.24)**	**2.02 (1.66, 2.46)**
Bullying victimization (ref. = no victimization)		
Being bullied at one time point	**1.45 (1.13, 1.84)**	**1.34 (1.07, 1.68)**
Being bullied at two time points	**1.62 (1.22, 2.13)**	**1.53 (1.18, 1.99)**
Any mood disorder diagnosis [*N* = 486]		
VP/VLBW birth (ref. = term‐born)	1.29 (0.90, 1.86)	1.38 (0.94, 2.05)
Bullying victimization (ref. = no victimization)		
Being bullied at one time point	1.40 (0.90, 2.18)	1.39 (0.89, 2.15)
Being bullied at two time points	**1.92 (1.17, 3.15)**	**2.08 (1.27, 3.42)**
Any anxiety disorder diagnosis [*N* = 486]		
VP/VLBW birth (ref. = term‐born)	1.06 (0.78, 1.44)	1.18 (0.86, 1.62)
Bullying victimization (ref. = no victimization)		
Being bullied at one time point	1.09 (0.75, 1.59)	1.10 (0.76, 1.60)
Being bullied at two time points	1.50 (0.98, 2.29)	**1.60 (1.05, 2.44)**

Negative binomial regression was used for internalizing symptoms given the skewed distribution and the overdispersion in the outcome. Robust Poisson regression was used for common binary outcomes (i.e., any mood disorder diagnosis, any anxiety disorder diagnosis) with prevalence rates ranging from 17.5% to 26.5%. We adjusted for sex, multiple birth, family socioeconomic status at birth, preexisting internalizing symptoms at age 6, and neurosensory impairment in childhood when analyzing the associations between bullying victimization and adult mental health, but only adjusted for sex, multiple birth, and family socioeconomic status at birth when analyzing the associations between VP/VLBW birth and adult mental health. Missing values in bullying victimization and confounders were imputed by chained equations. Bold fonts indicate statistically significant associations (*p* value < 0.05). CI, confidence interval; IRR, incidence rate ratio.

### Bullying victimization and mental health in adulthood across preterm and term‐born populations

Being bullied at two time points was associated with both self‐ and parent‐reported internalizing problems in adulthood. The significant associations remained after adjustments (self‐reported: IRR 1.61, 1.26–2.07; parent‐reported: IRR 1.53, 1.18–1.99). Similar results were shown for bullying victimization at one time point with internalizing problems with relatively smaller effect sizes than bullying at two time points (Table 2). Being bullied at two time points was positively associated with the likelihood of mood disorder diagnoses (adjusted IRR 2.08, 1.18–3.66; Table 2) and anxiety disorder diagnoses (adjusted IRR 1.60, 1.05–2.44). Results before imputation were overall consistent (Table S6). There were no significant interactive effects between VP/VLBW birth and bullying victimization (Table S7).

### Mediating role of bullying victimization

**Figure 3:**
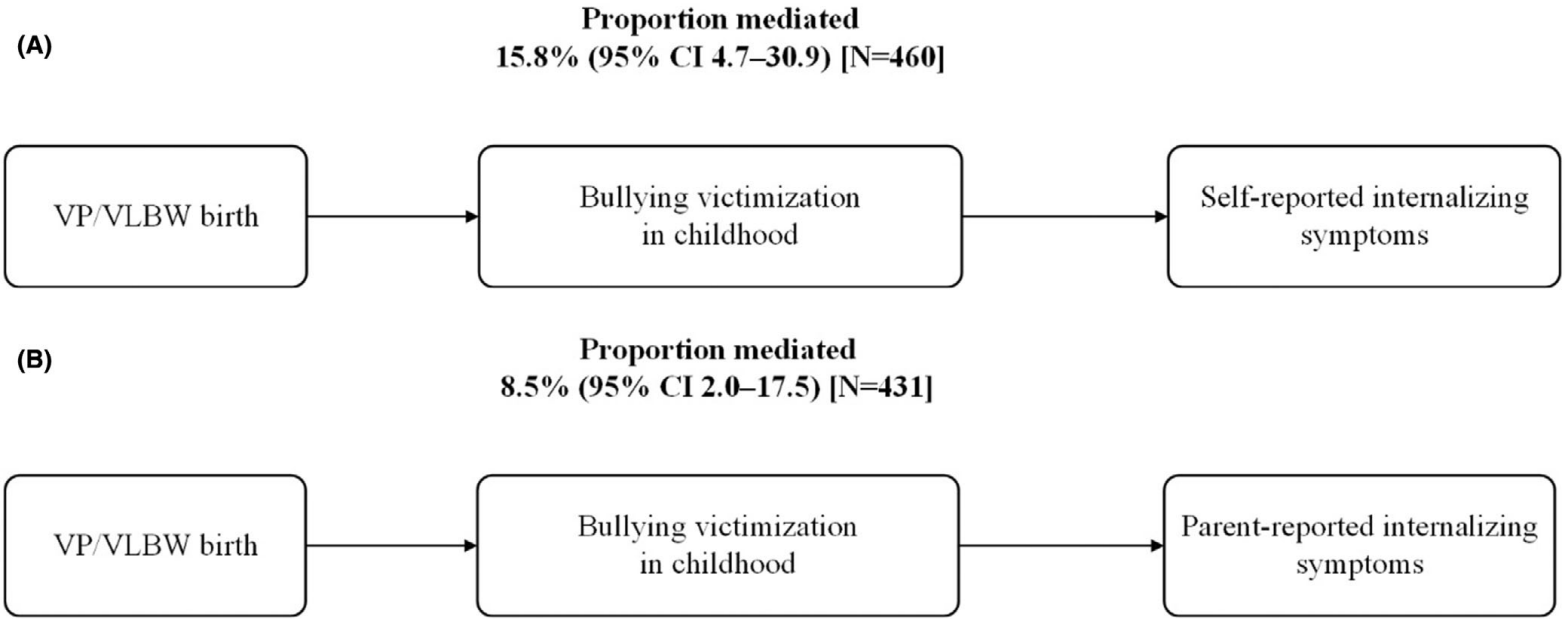
A causal mediation analysis of bullying victimization in childhood in the associations between VP/VLBW birth and adult mental health. A causal mediation analysis was performed in the combined sample of VP/VLBW and term‐born adults. The mediator was modeled using multinomial regression, and outcomes were modeled using negative binomial regression. Confounders unaffected by the exposure included sex, multiple birth, and family socioeconomic status at birth. Confounders affected by the exposure include preexisting internalizing symptoms at age 6 and neurosensory impairment in childhood. Missing values in the mediator and confounders were imputed. CI, confidence interval.

Individuals born VP/VLBW had increased risk for self‐ and parent‐reported internalizing symptoms compared with term‐born peers, which was partly explained by being bullied at school. Mediation analyses showed that bullying victimization mediated 15.8% (95% CI 4.7–30.9) of the effects of VP/VLBW birth on self‐reported internalizing symptoms (Figure 3; Figure S1). The sensitivity analysis indicated that to explain away the direct effect, the association of the unmeasured confounder with either the mediator or the outcome on risk ratio scale should take an *E*‐value of at least 1.15 (Table S8). For parent‐reported internalizing symptoms, bullying victimization mediated 8.5% (95% CI 2.0–17.5) of the effects of VP/VLBW birth (Figure 3; Figure S1). To explain away the direct effect, the association of the unmeasured confounder with either the mediator or the outcome on risk ratio scale should take an *E*‐value of at least 1.11 (Table S8). Results before imputation were consistent (Figure S2).

## Discussion

To the best of our knowledge, our study is the first to examine the moderating and mediating roles of bullying victimization in the association between preterm birth and mental health in adulthood. VP/VLBW participants were bullied more often in childhood and adolescence, and this partly explained that they had more internalizing symptoms in adulthood. Contrary to the preterm vulnerability hypothesis, bullying victimization did not moderate the associations between VP/VLBW birth and all mental health outcomes.

In line with previous research (Pyhala et al., 2017), we showed that VP/VLBW birth was significantly associated with increased internalizing symptoms in adulthood but not with mood and anxiety disorders. The distinction may arise from the fact that internalizing symptoms were assessed based on respondents' perceptions, potentially capturing subthreshold levels of mental health issues. In contrast, mood and anxiety disorders were diagnosed through structured diagnostic interviews, focusing on clinically significant syndromal levels of mental health concerns. Our results suggest that adults born VP/VLBW may exhibit heightened levels of worry, anxiety, and withdrawal, but they do not seem to have an elevated risk of developing clinically significant psychiatric disorders. This is consistent with a recent meta‐analysis of personality traits in adults born VP/VLBW indicating a heightened level of neuroticism (Liu et al., 2024).

Our results did not show that VP/VLBW participants were more vulnerable or sensitive to peer bullying than term‐born participants, contradicting a previous study using retrospective reports of bullying victimization which showed increased vulnerability to childhood adversity in ELBW individuals (Van Lieshout et al., 2018). However, in line with previous reports (Day et al., 2015; Grindvik et al., 2009; Nadeau et al., 2004; Wolke et al., 2015; Yau et al., 2013), those who were born VP/VLBW were bullied more often in childhood and adolescence. Our study also replicates previous research in that being bullied, in particular, chronically (more than one time point) was associated with increased internalizing symptoms and a higher risk of mental disorders (Lereya, Copeland, Costello, & Wolke, 2015; Moore et al., 2017). Our study further extends previous research by demonstrating that bullying victimization partly mediates the relationship between preterm birth and internalizing symptoms in adulthood (Liu et al., 2019). This has important implications as it indicates that the impact of preterm birth on mental health may be attenuated by interventions reducing bullying victimization. Despite no universally agreed‐upon standard for categorizing mediation effects as strong or weak, a proportion of 5%–10% is considered small in VanderWeele's widely cited book (VanderWeele, 2015). Following this, the proportions of 8.5% and 15.8% in our analyses could be considered small and moderate mediation, respectively. In psychology, it is often unrealistic to expect that a single mediator would fully explain the exposure–outcome association (MacKinnon, Fairchild, & Fritz, 2007). Future research should explore alternative modifiable mediators (e.g., overprotective parenting, quality of peer relationships, and friendship) (Day et al., 2018; Reyes, Jaekel, Bartmann, & Wolke, 2021) and perform mediation analysis with multiple mediators.

This study has several strengths. First, it is a 26‐year follow‐up study of a large whole population sample of VP/VLBW and term‐born individuals recruited in the same hospitals using prospective reports of bullying victimization and valid, reliable, and widely accepted measurement tools of mental health. Second, data on various neonatal and family factors and preexisting behavioral problems were available, allowing for the adjustment of confounders. Third, this study measured internalizing symptoms using both participant and parent‐reports and obtained diagnoses of mood and anxiety disorders through a structured interview conducted by trained psychologists. Lastly, we used multiple imputation to account for missing data in covariates. Imputed and original results were overall consistent, which strengthened our research findings.

There are also limitations. The first relates to the measure of bullying victimization. Information on school bullying at age 8 was obtained and assessed from parent report only. At age 13, it was collected from both participants and their parents, but only one general item from SDQ was utilized. Future research should collect data on bullying from multiple sources (e.g., participants themselves, friends, teachers) and utilize more specific items covering both direct and relational bullying. Second, 63% and 74% of eligible VP/VLBW and term‐born individuals were assessed at 26 years, larger retention than most new studies achieve at first recruitment nowadays (Kocar & Kaczmirek, 2024), and the dropout was associated with social disadvantage (Eryigit Madzwamuse et al., 2015). This is a common problem in long‐term cohort studies (Hille, Elbertse, Gravenhorst, Brand, & Verloove‐Vanhorick, 2005) and may affect group comparisons (Wolke et al., 2009). However, even when dropout is selective or correlated with the outcome, simulations have shown that predictions only marginally change (Wolke et al., 2009). Third, given the large confidence intervals in the mediation analysis, replication in a larger sample is required. Third, *E*‐values were reported as sensitivity analyses in our study to assess unmeasured confounding, which is often the central challenge in assessing evidence for causality in observational research. Despite no recommended threshold cut‐off, a large *E*‐value suggests that the observed mediation effect may be more robust to potential unmeasured confounding (VanderWeele & Ding, 2017). *E*‐values in our study may indicate that there is a possibility that the mediation result could be influenced by unmeasured variables that were not included in the analysis. Lastly, the findings of this study may be generalizable to similar populations, but caution should be exercised when extrapolating these results to populations with differing demographics or contexts. Future prospective studies involving VP/VLBW populations would benefit from incorporating assessments of childhood experiences, including bullying and other adversities, to enhance our understanding of the relationship between preterm birth, bullying victimization, and mental health.

In conclusion, being bullied has adverse effects on mental health in both VP/VLBW and term‐born children that last into adulthood. VP/VLBW participants were bullied more often in childhood and adolescence, and this partly explained that they had more internalizing symptoms in adulthood. The findings build on previous reports from this cohort (Wolke et al., 2015) and thus emphasize the need for interventions and support to help reduce peer bullying, which may reduce the burden of mental illness in adults in both preterm and total populations.

## Funding

This study was supported by grants from the German Federal Ministry of Education and Science (BMBF) PKE24, JUG14, 01EP9504, and 01ER0801. DW is supported by UK Research and Innovation (UKRI) under the UK government's Horizon Europe funding guarantee (ERC‐AdG award) [grant number EP/X023206/1]. The funders did not participate in the work.

## Conflict of interest

The authors have declared that they have no competing or potential conflicts of interest.

## Ethical considerations

Initial ethical approval was obtained from the Ethics Committee of the University of Munich Children's Hospital and the Bavarian Health Council. Ethical approval for the follow‐up in adulthood was obtained from the Ethical Board of the University Hospital Bonn (reference 159/09). Parents provided informed written consent within 48 hr of their child's birth and all participants provided informed written consent for the assessments in adulthood. In case of severe impairment of the adult participant, consent was provided by an assigned guardian (usually a parent). The STROBE guidelines were used to ensure the reporting of this cohort study (Table S1).

## Supporting information


**Figure S1.** A causal mediation analysis of bullying victimization in childhood in the associations between VP/VLBW birth and adult mental health (imputed results).
**Figure S2.** A causal mediation analysis of bullying victimization in childhood in the associations between VP/VLBW birth and adult mental health (complete case analysis).
**Table S1.** STROBE Statement—Checklist of items that should be included in reports of cohort studies.
**Table S2.** Subtypes of DSM‐IV mood and anxiety disorders.
**Table S3.** Model fit statistics for associations of VP/VLBW birth and bullying victimization with internalizing symptoms (complete case analysis).
**Table S4.** Model fit statistics for associations of VP/VLBW birth and bullying victimization with mood or anxiety disorder diagnoses (complete case analysis).
**Table S5.** Model fit statistics for the mediation analysis (complete case analysis).
**Table S6.** Associations of VP/VLBW birth and bullying victimization with mental health (complete case analysis).
**Table S7.** Interaction between VP/VLBW birth and bullying victimization in relation to adult mental health.
**Table S8.** Sensitivity analysis results for unmeasured confounding in the mediation analysis.
**Appendix S1.** Stata codes for the association analysis.
**Appendix S2.** R codes for the causal mediation analysis.

## Data Availability

Data can be requested for research purposes and will require a data transfer agreement.
