# Supplementary material for: Preterm birth, bullying victimization, and mental health in adulthood: A prospective cohort study in Germany
Source: Child Adolesc Ment Health. 2025 Aug 8;30(4):343–51. doi: 10.1111/camh.70025 (PMC12573058; doi:10.1111/camh.70025)
Supplement: Supplementary file 1 — Figure S1. A causal mediation analysis of bullying victimization in childhood in the associations between VP/VLBW birth and adult mental health (imputed results). Figure S2. A causal mediation analysis of bullying victimization in childhood in the associations between VP/VLBW birth and adult mental health (complete case analysis). Table S1. STROBE Statement—Checklist of items that should be included in reports of cohort studies. Table S2. Subtypes of DSM‐IV mood and anxiety disorders. Table S3. Model fit statistics for associations of VP/VLBW birth and bullying victimization with internalizing symptoms (complete case analysis). Table S4. Model fit statistics for associations of VP/VLBW birth and bullying victimization with mood or anxiety disorder diagnoses (complete case analysis). Table S5. Model fit statistics for the mediation analysis (complete case analysis). Table S6. Associations of VP/VLBW birth and bullying victimization with mental health (complete case analysis). Table S7. Interaction between VP/VLBW birth and bullying victimization in relation to adult mental health. Table S8. Sensitivity analysis results for unmeasured confounding in the mediation analysis. Appendix S1. Stata codes for the association analysis. Appendix S2. R codes for the causal mediation analysis. [file CAMH-30-343-s001.docx]

**Supplementary Materials**

[Figure S1 A causal mediation analysis of bullying victimization in childhood in the associations between VP/VLBW birth and adult mental health (imputed results) 2](#_Toc190340968)

[Figure S2 A causal mediation analysis of bullying victimization in childhood in the associations between VP/VLBW birth and adult mental health (complete case analysis) 3](#_Toc190340969)

[Table S1 STROBE Statement—Checklist of items that should be included in reports of cohort studies 4](#_Toc190340970)

[Table S2 Subtypes of DSM-IV mood and anxiety disorders 6](#_Toc190340971)

[Table S3 Model fit statistics for associations of VP/VLBW birth and bullying victimization with internalizing symptoms (complete case analysis) 7](#_Toc190340972)

[Table S4 Model fit statistics for associations of VP/VLBW birth and bullying victimization with mood or anxiety disorder diagnoses (complete case analysis) 8](#_Toc190340973)

[Table S5 Model fit statistics for the mediation analysis (complete case analysis) 9](#_Toc190340974)

[Table S6 Associations of VP/VLBW birth and bullying victimization with mental health (complete case analysis) 10](#_Toc190340975)

[Table S7 Interaction between VP/VLBW birth and bullying victimization in relation to adult mental health 11](#_Toc190340976)

[Table S8 Sensitivity analysis results for unmeasured confounding in the mediation analysis 12](#_Toc190340977)

[Stata codes for the association analysis 13](#_Toc190340978)

[R codes for the causal mediation analysis 16](#_Toc190340979)

Figure S1 A causal mediation analysis of bullying victimization in childhood in the associations between VP/VLBW birth and adult mental health (imputed results)

**a.** Self-reported internalizing symptoms as the outcome

**
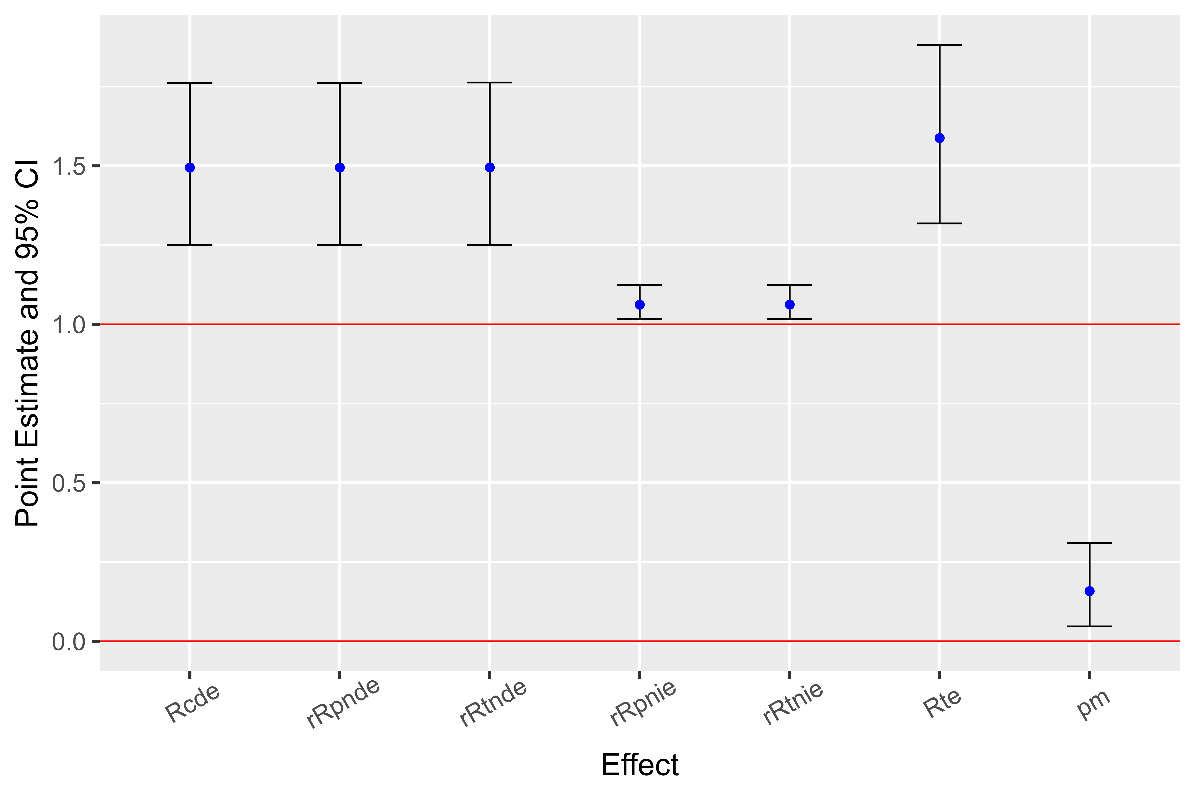
**

**b.** Parent-reported internalizing symptoms as the outcome

**
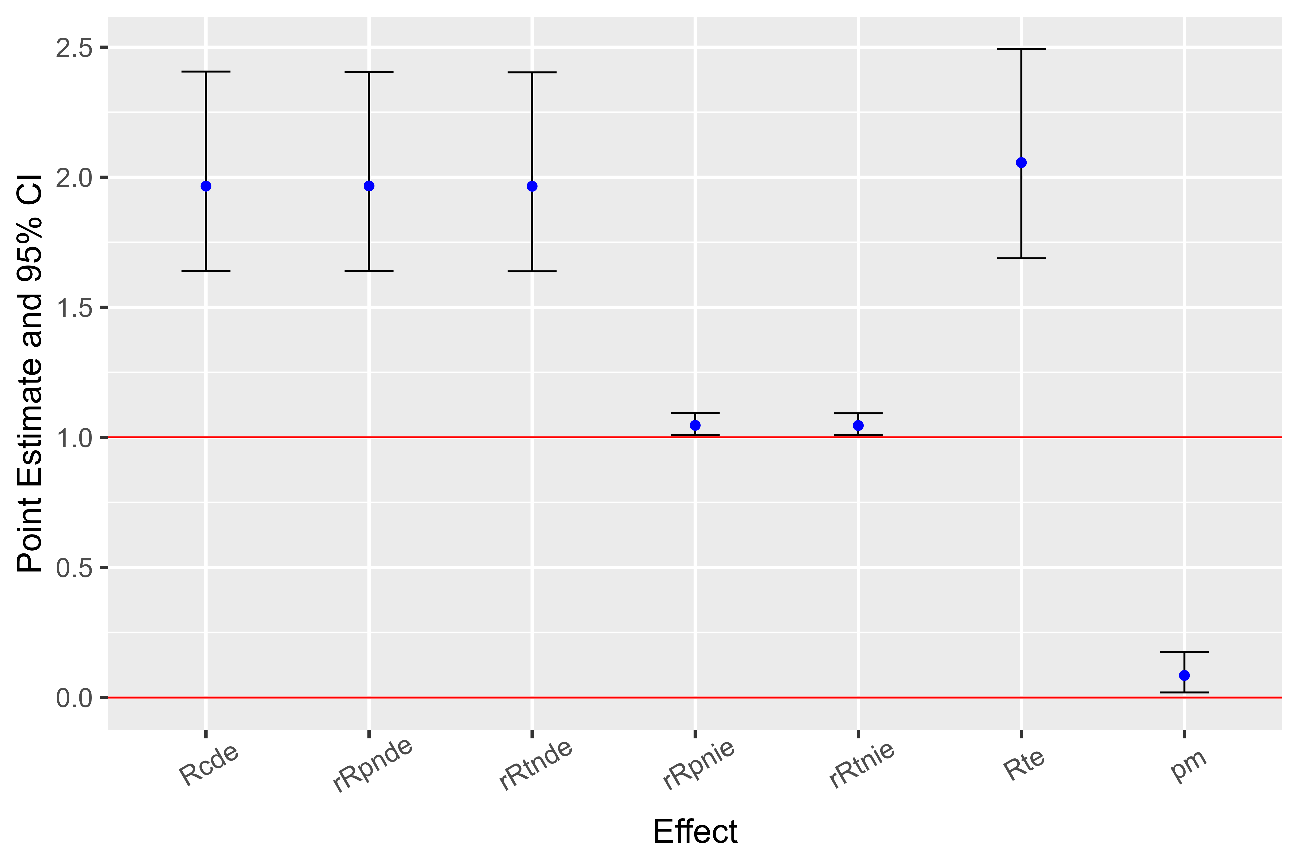
**

Abbreviation: CI, confidence interval; Rcde, controlled direct effect rate ratio; rRpnde, randomized analogue of pure natural direct effect rate ratio; rRtnde, randomized analogue of total natural direct effect rate ratio; rRpnie, randomized analogue of pure natural indirect effect rate ratio; rRtnie, randomized analogue of total natural indirect effect rate ratio; Rte, total effect rate ratio; rpm, randomized analogue of overall proportion mediated.

Figure S2 A causal mediation analysis of bullying victimization in childhood in the associations between VP/VLBW birth and adult mental health (complete case analysis)

**a.** Self-reported internalizing symptoms as the outcome

**
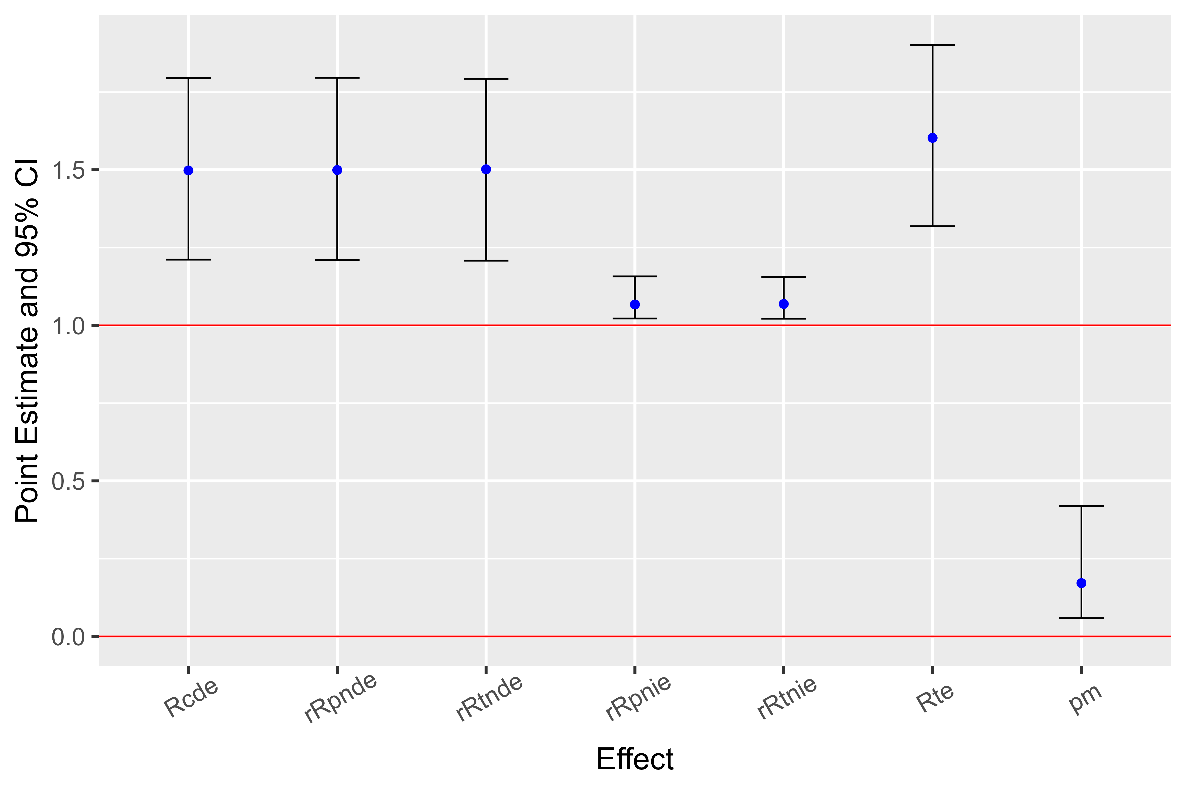
**

**b.** Parent-reported internalizing symptoms as the outcome

**
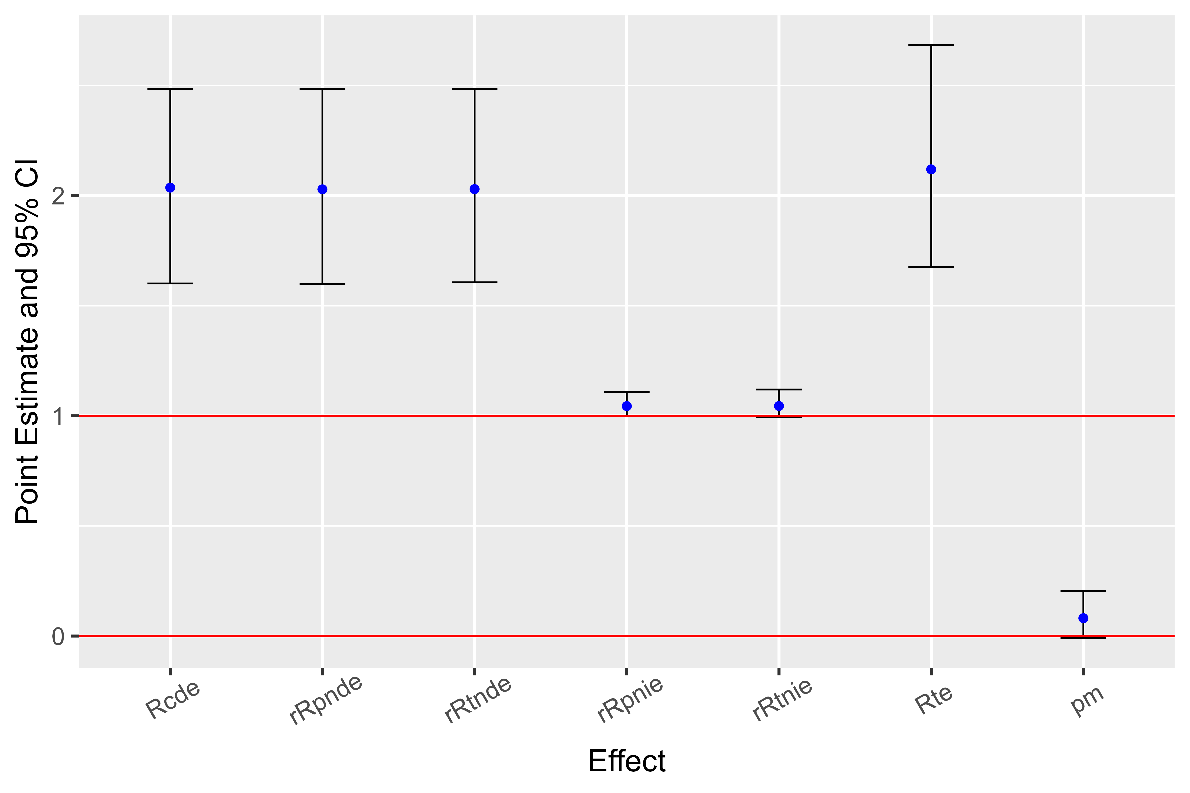
**

Abbreviation: CI, confidence interval; Rcde, controlled direct effect rate ratio; rRpnde, randomized analogue of pure natural direct effect rate ratio; rRtnde, randomized analogue of total natural direct effect rate ratio; rRpnie, randomized analogue of pure natural indirect effect rate ratio; rRtnie, randomized analogue of total natural indirect effect rate ratio; Rte, total effect rate ratio; rpm, randomized analogue of overall proportion mediated.

Table S1 STROBE Statement—Checklist of items that should be included in reports of cohort studies

|  | **Item No** | **Recommendation** | **Page No** |
| --- | --- | --- | --- |
| **Title and abstract** | 1 | (*a*) Indicate the study’s design with a commonly used term in the title or the abstract | Title page |
|  |  | (*b*) Provide in the abstract an informative and balanced summary of what was done and what was found | 1 |
| **Introduction** | | | |
| Background/rationale | 2 | Explain the scientific background and rationale for the investigation being reported | 3-4 |
| Objectives | 3 | State specific objectives, including any prespecified hypotheses | 4-5 |
| **Methods** | | | |
| Study design | 4 | Present key elements of study design early in the paper | 5 |
| Setting | 5 | Describe the setting, locations, and relevant dates, including periods of recruitment, exposure, follow-up, and data collection | 5 |
| Participants | 6 | (*a*) Give the eligibility criteria, and the sources and methods of selection of participants. Describe methods of follow-up | 5 |
|  |  | (*b*) For matched studies, give matching criteria and number of exposed and unexposed |  |
| Variables | 7 | Clearly define all outcomes, exposures, predictors, potential confounders, and effect modifiers. Give diagnostic criteria, if applicable | 6-8 |
| Data sources/ measurement | 8* | For each variable of interest, give sources of data and details of methods of assessment (measurement). Describe comparability of assessment methods if there is more than one group | 6-8 |
| Bias | 9 | Describe any efforts to address potential sources of bias | 8-9 |
| Study size | 10 | Explain how the study size was arrived at | 5 |
| Quantitative variables | 11 | Explain how quantitative variables were handled in the analyses. If applicable, describe which groupings were chosen and why | 6-8 |
| Statistical methods | 12 | (*a*) Describe all statistical methods, including those used to control for confounding | 8-9 |
|  |  | (*b*) Describe any methods used to examine subgroups and interactions | 8 |
|  |  | (*c*) Explain how missing data were addressed | 8-9 |
|  |  | (*d*) If applicable, explain how loss to follow-up was addressed | 13 |
|  |  | (*e*) Describe any sensitivity analyses | 8-9 |
| **Results** | | |  |
| Participants | 13* | (a) Report numbers of individuals at each stage of study—eg numbers potentially eligible, examined for eligibility, confirmed eligible, included in the study, completing follow-up, and analysed | 9 |
|  |  | (b) Give reasons for non-participation at each stage | 5 |
|  |  | (c) Consider use of a flow diagram | 5 |
| Descriptive data | 14* | (a) Give characteristics of study participants (eg demographic, clinical, social) and information on exposures and potential confounders | 9 |
|  |  | (b) Indicate number of participants with missing data for each variable of interest | 9 |
|  |  | (c) Summarise follow-up time (eg, average and total amount) | 5 |
| Outcome data | 15* | Report numbers of outcome events or summary measures over time | 9 |

| Main results | 16 | (*a*) Give unadjusted estimates and, if applicable, confounder-adjusted estimates and their precision (eg, 95% confidence interval). Make clear which confounders were adjusted for and why they were included | 10-11 |
| --- | --- | --- | --- |
|  |  | (*b*) Report category boundaries when continuous variables were categorized | NA |
|  |  | (*c*) If relevant, consider translating estimates of relative risk into absolute risk for a meaningful time period | NA |
| Other analyses | 17 | Report other analyses done—eg analyses of subgroups and interactions, and sensitivity analyses | 10-11 |
| **Discussion** | | | |
| Key results | 18 | Summarise key results with reference to study objectives | 11 |
| Limitations | 19 | Discuss limitations of the study, taking into account sources of potential bias or imprecision. Discuss both direction and magnitude of any potential bias | 13 |
| Interpretation | 20 | Give a cautious overall interpretation of results considering objectives, limitations, multiplicity of analyses, results from similar studies, and other relevant evidence | 11-14 |
| Generalisability | 21 | Discuss the generalisability (external validity) of the study results | 13-14 |
| **Other information** | | | |
| Funding | 22 | Give the source of funding and the role of the funders for the present study and, if applicable, for the original study on which the present article is based | 14 |

*Give information separately for exposed and unexposed groups.

**Note:** An Explanation and Elaboration article discusses each checklist item and gives methodological background and published examples of transparent reporting. The STROBE checklist is best used in conjunction with this article (freely available on the Web sites of PLoS Medicine at http://www.plosmedicine.org/, Annals of Internal Medicine at http://www.annals.org/, and Epidemiology at http://www.epidem.com/). Information on the STROBE Initiative is available at http://www.strobe-statement.org.

Table S2 Subtypes of DSM-IV mood and anxiety disorders

|  | **Subtypes** |
| --- | --- |
| **Any mood disorder** | Mood disorder due to a general medical condition |
|  | Substance-induced mood disorder |
|  | Manic episode |
|  | Hypomanic episode |
|  | Major depressive episode |
|  | Mixture of manic episode and major depressive episode |
|  | Bipolar 1 disorder |
|  | Bipolar 2 disorder |
|  | Cyclothymic disorder |
|  | Bipolar disorder not otherwise specified |
|  | Major depressive disorder |
|  | Dsythymic disorder |
|  | Mood disorder with depressive features |
| **Any anxiety disorder** | Panic attack |
|  | Agoraphobia |
|  | Agoraphobia w/o. panic attacks |
|  | Panic disorder with agoraphobia |
|  | Panic disorder w/o. agoraphobia |
|  | Social phobia |
|  | Specific phobia, animal type |
|  | Specific phobia, nat. env. Type |
|  | Specific phobia, blood-injection |
|  | Specific phobia, situational type |
|  | Specific phobia, other type |
|  | Phobic disorder NOS (not otherwise specified) |
|  | Generalized anxiety disorder |
|  | GMC anxiety disorder (due to a general medical condition) |

Table S3 Model fit statistics for associations of VP/VLBW birth and bullying victimization with internalizing symptoms (complete case analysis)

|  | **Internalizing symptoms (self-report)** |
| --- | --- |
| VP/VLBW birth as the exposure |  |
| *Log Likelihood* | -1424.4 |
| *Likelihood Ratio chi2(5)* | 28.8 |
| *Prob > chi2* | <0.001 |
| *Pseudo R^2^* | 0.010 |
| *Likelihood Ratio test of alpha=0* | <0.001 |
| Bullying victimization as the exposure |  |
| *Log Likelihood* | -1217.5 |
| *Likelihood Ratio chi2(8)* | 27.55 |
| *Prob > chi2* | 0.001 |
| *Pseudo R^2^* | 0.011 |
| *Likelihood Ratio test of alpha=0* | <0.001 |
|  | **Internalizing symptoms (parent report)** |
|  |  |
| VP/VLBW birth as the exposure |  |
| *Log Likelihood* | -1156.6 |
| *Likelihood Ratio chi2(5)* | 49.7 |
| *Prob > chi2* | <0.001 |
| *Pseudo R^2^* | 0.021 |
| *Likelihood Ratio test of alpha=0* | <0.001 |
| Bullying victimization as the exposure |  |
| *Log Likelihood* | -963.9 |
| *Likelihood Ratio chi2(8)* | 54.77 |
| *Prob > chi2* | <0.001 |
| *Pseudo R^2^* | 0.028 |
| *Likelihood Ratio test of alpha=0* | <0.001 |

Negative binomial regression was used for internalizing symptoms given the skewed distribution and the over-dispersion in the outcome. We adjusted for sex, multiple birth, family socioeconomic status at birth, pre-existing internalizing symptoms at age 6, and neurosensory impairment in childhood when analyzing the associations between bullying victimization and adult mental health, but only adjusted for sex, multiple birth, and family socioeconomic status at birth when analyzing the associations between VP/VLBW birth and adult mental health.

Table S4 Model fit statistics for associations of VP/VLBW birth and bullying victimization with mood or anxiety disorder diagnoses (complete case analysis)

|  | **Any mood disorder diagnosis** |
| --- | --- |
| VP/VLBW birth as the exposure |  |
| *Log pseudolikelihood* | -248.7 |
| *Wald chi2(5)* | 16.1 |
| *Prob > chi2* | 0.007 |
| *Pseudo R^2^* | 0.024 |
| *Deviance goodness-of-fit test P value* | 1.000 |
| Bullying victimization as the exposure |  |
| *Log pseudolikelihood* | -210.6 |
| *Wald chi2(8)* | 25.2 |
| *Prob > chi2* | 0.001 |
| *Pseudo R^2^* | 0.039 |
| *Deviance goodness-of-fit test P value* | 1.000 |
|  | **Any anxiety disorder diagnosis** |
|  |  |
| VP/VLBW birth as the exposure |  |
| *Log pseudolikelihood* | -289.6 |
| *Wald chi2(5)* | 14.9 |
| *Prob > chi2* | 0.011 |
| *Pseudo R^2^* | 0.017 |
| *Deviance goodness-of-fit test P value* | 1.000 |
| Bullying victimization as the exposure |  |
| *Log pseudolikelihood* | -244.9 |
| *Wald chi2(8)* | 13.2 |
| *Prob > chi2* | 0.105 |
| *Pseudo R^2^* | 0.018 |
| *Deviance goodness-of-fit test P value* | 1.000 |

Robust Poisson regression was used for common binary outcomes (i.e., any mood disorder diagnosis, any anxiety disorder diagnosis) with prevalence rates ranging from 17.5-26.5%. We adjusted for sex, multiple birth, family socioeconomic status at birth, pre-existing internalizing symptoms at age 6, and neurosensory impairment in childhood when analyzing the associations between bullying victimization and adult mental health, but only adjusted for sex, multiple birth, and family socioeconomic status at birth when analyzing the associations between VP/VLBW birth and adult mental health.

Table S5 Model fit statistics for the mediation analysis (complete case analysis)

|  | **Internalizing symptoms (self-report)** | |
| --- | --- | --- |
|  | **Outcome regression** | **Mediator regression** |
| Residual Deviance | 448.3 | 783.2 |
| AIC | 2445 | 815.2 |
|  | **Internalizing symptoms (parent-report)** | |
|  | **Outcome regression** | **Mediator regression** |
| Residual Deviance | 414.9 | 728.1 |
| AIC | 1925.1 | 760.1 |

The mediator was modeled using multinomial regression and outcomes were modeled using negative binomial regression.

Table S6 Associations of VP/VLBW birth and bullying victimization with mental health (complete case analysis)

|  | **Internalizing symptoms (self-report)** | |
| --- | --- | --- |
|  | Unadjusted | Adjusted |
|  | IRR (95% CI) | IRR (95% CI) |
| VP/VLBW birth (ref.=term-born) | **1.43 (1.20, 1.70) [N=460]** | **1.58 (1.31, 1.90) [N=459]** |
| Bullying victimization (ref.=no victimization) |  |  |
| *Being bullied at one time point* | **1.28 (1.04, 1.58) [N=395]** | 1.22 (0.99, 1.51) [N=393] |
| *Being bullied at two time points* | **1.73 (1.33, 2.25) [N=395]** | **1.65 (1.27, 2.16) [N=393]** |
|  | **Internalizing symptoms (parent report)** | |
|  | Unadjusted | Adjusted |
|  | IRR (95% CI) | IRR (95% CI) |
| VP/VLBW birth (ref.=term-born) | **1.86 (1.55, 2.24) [N=431]** | **2.03 (1.67, 2.47) [N=430]** |
| Bullying victimization (ref.=no victimization) |  |  |
| *Being bullied at one time point* | **1.34 (1.08, 1.68) [N=409]** | **1.33 (1.06, 1.66) [N=366]** |
| *Being bullied at two time points* | **1.75 (1.33, 2.31) [N=409]** | **1.55 (1.17, 2.05) [N=366]** |
|  | **Any mood disorder diagnosis** | |
|  | Unadjusted | Adjusted |
|  | IRR (95% CI) | IRR (95% CI) |
| VP/VLBW birth (ref.=term-born) | 1.29 (0.90, 1.86) [N=486] | 1.39 (0.94, 2.05) [N=485] |
| Bullying victimization (ref.=no victimization) |  |  |
| *Being bullied at one time point* | 1.39 (0.89, 2.16) [N=407] | 1.34 (0.87, 2.08) [N=405] |
| *Being bullied at two time points* | **1.91 (1.18, 3.08) [N=407]** | **2.05 (1.28, 3.30) [N=405]** |
|  | **Any anxiety disorder diagnosis** | |
|  | Unadjusted | Adjusted |
|  | IRR (95% CI) | IRR (95% CI) |
| VP/VLBW birth (ref.=term-born) | 1.06 (0.78, 1.44) [N=486] | 1.18 (0.86, 1.62) [N=485] |
| Bullying victimization (ref.=no victimization) |  |  |
| *Being bullied at one time point* | 1.09 (0.75, 1.58) [N=407] | 1.08 (0.74, 1.58) [N=405] |
| *Being bullied at two time points* | 1.48 (0.98, 2.23) [N=407] | **1.52 (1.00, 2.29) [N=405]** |

Negative binomial regression was used for internalizing symptoms given the skewed distribution and the over-dispersion in the outcome. Robust Poisson regression was used for common binary outcomes (i.e., any mood disorder diagnosis, any anxiety disorder diagnosis) with prevalence rates ranging from 17.5-26.5%. We adjusted for sex, multiple birth, family socioeconomic status at birth, pre-existing internalizing symptoms at age 6, and neurosensory impairment in childhood when analyzing the associations between bullying victimization and adult mental health, but only adjusted for sex, multiple birth, and family socioeconomic status at birth when analyzing the associations between VP/VLBW birth and adult mental health. Abbreviations: CI, confidence interval; IRR, Incidence Rate Ratio.

Table S7 Interaction between VP/VLBW birth and bullying victimization in relation to adult mental health

|  | **Self-reported internalizing symptoms [N=460]** | **Parent-reported internalizing symptoms [N=431]** |
| --- | --- | --- |
|  | IRR (95% CI) | IRR (95% CI) |
| VP/VLBW birth (ref.=term-born) | 1.57 (1.18, 2.10) | 1.82 (1.35, 2.45)] |
| Bullying victimization (ref.=no victimization) |  |  |
| *Being bullied at one time point* | 1.27 (0.97, 1.66) | 1.30 (0.97, 1.74) |
| *Being bullied at two time points* | 1.67 (1.13, 2.47) | 1.78 (1.18, 2.69) |
| VP/VLBW birth * bullying victimization |  |  |
| *VP/VLBW birth *being bullied at one time point* | 0.88 (0.59, 1.30) | 1.00 (0.66, 1.53) |
| *VP/VLBW birth *being bullied at two time points* | 0.86 (0.51, 1.44) | 0.70 (0.41, 1.20) |
|  | **Any mood disorder diagnosis [N=486]** | **Any anxiety disorder diagnosis [N=486]** |
|  | IRR (95% CI) | IRR (95% CI) |
| VP/VLBW birth (ref.=term-born) | 1.75 (0.88, 3.48) | 1.04 (0.61, 1.79) |
| Bullying victimization (ref.=no victimization) |  |  |
| *Being bullied at one time point* | 1.67 (0.87, 3.21) | 0.88 (0.52, 1.48) |
| *Being bullied at two time points* | 2.56 (1.21, 5.40) | 1.66 (0.97, 2.84) |
| VP/VLBW birth * bullying victimization |  |  |
| *VP/VLBW birth *being bullied at one time point* | 0.70 (0.29, 1.70) | 1.53 (0.72, 3.26) |
| *VP/VLBW birth *being bullied at two time points* | 0.65 (0.25, 1.72) | 0.94 (0.42, 2.13) |

Negative binomial regression was used for internalizing symptoms given the skewed distribution and the over-dispersion in the outcome. Robust Poisson regression was used for common binary outcomes (i.e., any mood disorder diagnosis, any anxiety disorder diagnosis) with prevalence rates ranging from 17.5-26.5%. Models adjusted for sex, multiple birth, family socioeconomic status at birth, pre-existing internalizing symptoms at age 6, and neurosensory impairment in childhood. Missing values in bullying victimization and confounders were imputed by chained equations. Abbreviations: CI, confidence interval; IRR, Incidence Rate Ratio.

Table S8 Sensitivity analysis results for unmeasured confounding in the mediation analysis

|  | **Self-reported internalizing symptoms** | | **Parent-reported internalizing symptoms** | |
| --- | --- | --- | --- | --- |
|  | **E-value** | **95% CI Lower Bound** | **E-value** | **95% CI Lower Bound** |
| Rcde | 2.35 | 1.81 | 3.34 | 2.66 |
| rRpnde | 2.35 | 1.81 | 3.34 | 2.66 |
| rRtnde | 2.36 | 1.81 | 3.34 | 2.66 |
| rRpnie | 1.32 | 1.15 | 1.27 | 1.10 |
| rRtnie | 1.32 | 1.15 | 1.26 | 1.11 |
| Rte | 2.55 | 1.97 | 3.53 | 2.77 |

Abbreviation: CI, confidence interval; Rcde, controlled direct effect rate ratio; rRpnde, randomized analogue of pure natural direct effect rate ratio; rRtnde, randomized analogue of total natural direct effect rate ratio; rRpnie, randomized analogue of pure natural indirect effect rate ratio; rRtnie, randomized analogue of total natural indirect effect rate ratio; Rte, total effect rate ratio.

Stata codes for the association analysis

**Four mental health outcomes

local myoutlist internalising pinternalising Any_mood_disorder Any_anxiety_disorder

foreach myout in `myoutlist' {

use "BLS_bullying2.dta", clear

keep if !missing(`myout') & !missing(groupP4)

keep `myout' blsgroup victim_8y_13ypc gender mul_birth ses0y2 nsi i

save BLS_bullying2_`myout'.dta, replace

}

***self-reported internalizing symptoms- create imputed datasets

use BLS_bullying2_internalising.dta,clear

*Let's summarize missing values

misstable summarize, generate(Mis_)

*and explore missing-data patterns

misstable patterns

*Declare the storage style

mi set mlong

*Register variables

mi register imputed ses0y2 nsi i victim_8y_13ypc

mi register regular gender mul_birth blsgroup internalising

*multiple imputation

mi impute chained (logit) nsi (mlogit) ses0y2 victim_8y_13ypc (regress) i = i.gender i.mul_birth i.blsgroup internalising, add(20) burnin(10) savetrace(impstats_e1, replace) rseed(27654) noisily augment

save BLS_bullying2_internalising_20mi_sen.dta,replace

***Same codes were used for other outcomes to create imputed datasets

*****Model estimates for internalizing symptoms**

*VP/VLBW birth as the exposure

local myoutlist internalising pinternalising

foreach myout in `myoutlist' {

use BLS_bullying2_`myout'_20mi_sen.dta, clear

local myvarlist blsgroup

foreach myvar in `myvarlist' {

mi estimate: nbreg `myout' ib2.`myvar'

mi estimate: nbreg `myout' ib2.`myvar' i.gender i.mul_birth i.ses0y2

}/*close of the myvar loop*/

}/*close of the myout loop*/

*Bullying victimization as the exposure

local myoutlist internalising pinternalising

foreach myout in `myoutlist' {

use BLS_bullying2_`myout'_20mi_sen.dta, clear

local myvarlist victim_8y_13ypc

foreach myvar in `myvarlist' {

mi estimate: nbreg `myout' i.`myvar'

mi estimate: nbreg `myout' i.`myvar' i.gender i.mul_birth i.ses0y2 i.nsi i

}/*close of the myvar loop*/

}/*close of the myout loop*/

*****Model estimates for diagnoses of mental disorders**

*VP/VLBW birth as the exposure

local myoutlist Any_mood_disorder Any_anxiety_disorder

foreach myout in `myoutlist' {

use BLS_bullying2_`myout'_20mi_sen.dta, clear

local myvarlist blsgroup

foreach myvar in `myvarlist' {

mi estimate: poisson `myout' ib2.`myvar' , vce(robust)

mi estimate: poisson `myout' ib2.`myvar' i.gender i.mul_birth i.ses0y2 , vce(robust)

}/*close of the myvar loop*/

}/*close of the myout loop*/

*Bullying victimization as the exposure

local myoutlist Any_mood_disorder Any_anxiety_disorder

foreach myout in `myoutlist' {

use BLS_bullying2_`myout'_20mi_sen.dta, clear

local myvarlist victim_8y_13ypc

foreach myvar in `myvarlist' {

mi estimate: glm `myout' i.`myvar', family(poisson) link(log) eform vce(robust)

mi estimate: glm `myout' i.`myvar' i.gender i.mul_birth i.ses0y2 i.nsi i , family(poisson) link(log) eform vce(robust)

}/*close of the myvar loop*/

}/*close of the myout loop*/

*****Interaction**

***Model estimates for internalizing symptoms

local myoutlist internalising pinternalising

foreach myout in `myoutlist' {

use BLS_bullying2_`myout'_20mi_sen.dta, clear

mi estimate: nbreg `myout' ib2.blsgroup##victim_8y_13ypc i.gender i.mul_birth i.ses0y2 i.nsi i

}/*close of the myout loop*/

***Model estimates for diagnoses of mental disorders

local myoutlist Any_mood_disorder Any_anxiety_disorder

foreach myout in `myoutlist' {

use BLS_bullying2_`myout'_20mi_sen.dta, clear

mi estimate: glm `myout' ib2.blsgroup##victim_8y_13ypc i.gender i.mul_birth i.ses0y2 i.nsi i, family(poisson) link(log) vce(robust)

}/*close of the myout loop*/

R codes for the causal mediation analysis

##Codes are the same for self- and parent-reported internalizing symptoms

##install CMAverse

devtools::install_github("BS1125/CMAverse")

library(CMAverse)

library(writexl)

##Read CSV file

med1 <-read.table("mediation_internalising5.csv",sep = ",", header = TRUE, quote = "\"")

med1<- as.data.frame(med1)

names(med1)

names <- c(2:7)

med1[,names] <- lapply(med1[,names] , factor)

## Plot the directed acyclic graph (DAG) for causal mediation analysis

cmdag(outcome = "internalising",

exposure = "blsgroup_yn",

mediator = c("victim_8y_13ypc"),

basec = c("male","mul_birth","ses0y2"),

postc = c("nsi","i"), node = FALSE, text_col = "black")

## Causal mediation analysis using the g-formula approach

set.seed(2014)

med1_est <- cmest(data = med1, model = "gformula", outcome = "internalising",

exposure = "blsgroup_yn",

mediator = c("victim_8y_13ypc"),

basec = c("male","mul_birth","ses0y2"),

postc = c("nsi","i"),

EMint = FALSE,

mreg = list("multinomial"),

yreg = "negbin",

postcreg = list("logistic","negbin"),

astar = 0, a = 1, mval = list("victim_8y_13ypc_0"),

multimp = TRUE, args_mice = list(m = 20),

estimation = "imputation", inference = "bootstrap",

nboot = 200, boot.ci.type = "per")

summary(med1_est)

library(ggplot2)

ggcmest(med1_est) +

ggplot2::theme(axis.text.x = ggplot2::element_text(angle = 30, vjust = 0.8))

##sensitivity analyses

med1_est_sens <- cmsens(object = med1_est, sens = "uc")

med1_est_sens
